# Exploring Splicing Modulation as an Innovative Approach to Combat Pancreatic Cancer: SF3B1 Emerges as a Prognostic Indicator and Therapeutic Target

**DOI:** 10.7150/ijbs.92671

**Published:** 2024-06-03

**Authors:** Rocco Sciarrillo, Francesca Terrana, Annalisa Comandatore, I Gede Putu Supadmanaba, Bing Wang, Btissame El Hassouni, Giulia Mantini, Gerrit Jansen, Amir Avan, Daniela Carbone, Patrizia Diana, Godefridus J Peters, Luca Morelli, Jacqueline Cloos, Yehuda G Assaraf, Elisa Giovannetti

**Affiliations:** 1 Department of Medical Oncology, Cancer Center Amsterdam, VU University Medical Center, Amsterdam, The Netherlands.; 2 Department of Pediatric Oncology, Cancer Center Amsterdam, VU University Medical Center, Amsterdam, The Netherlands.; 3 Department of Hematology, Cancer Center Amsterdam, VU University Medical Center, Amsterdam, The Netherlands.; 4 Dipartimento di Scienze e Tecnologie Biologiche Chimiche e Farmaceutiche (STEBICEF), Università degli Studi di Palermo, Palermo, Italy.; 5 General Surgery Unit, Department of Translational Research and New Technologies in Medicine and Surgery, University of Pisa, Pisa, Italy.; 6 Biochemistry Department, Faculty of Medicine, Universitas Udayana, Denpasar, Bali, Indonesia.; 7 Cancer Pharmacology Lab, Fondazione Pisana per la Scienza, Pisa, Italy.; 8 Amsterdam Rheumatology and immunology Center, VU University Medical Center, Amsterdam, The Netherlands.; 9 Metabolic Syndrome Research Center, Mashhad University of Medical Sciences, Mashhad, Iran.; 10 Faculty of Health, School of Biomedical Sciences, Queensland University of Technology, Brisbane, Australia.; 11 Department of Biochemistry, Medical University of Gdansk, Gdańsk, Poland.; 12 Fred Wyszkowski Cancer Research Laboratory, Department of Biology, Technion-Israel Institute of Technology, Haifa, Israel.

**Keywords:** PDAC, splicing, SF3B1, SF3B1 modulators, Pladienolide-B, E7107.

## Abstract

Pancreatic ductal adenocarcinoma (PDAC) poses significant challenges in terms of prognosis and treatment. Recent research has identified splicing deregulation as a new cancer hallmark. Herein, we investigated the largely uncharacterized alternative splicing profile and the key splicing factor SF3B1 in PDAC pancreatic cells and tissues as a potential discovery source of plausible drug targets and new predictive biomarkers of clinical outcome. The research involved a transcriptome-wide analysis, comparing profiles of splicing profiles in PDAC primary cells with normal ductal cells. This revealed more than 400 significant differential splicing events in genes involved in regulation of gene expression, primarily related to mRNA splicing, and metabolism of nucleic acids. PDAC cultures were highly sensitive to the SF3B1 modulators, E7107 and Pladienolide-B, showing IC50s in the low nanomolar range. These compounds induced apoptosis, associated to induction of the MCL-1/S splice variant. and reduced cell migration, associated to RON mis-splicing. In an orthotopic mouse model, E7107 showed promising results. Furthermore, we evaluated SF3B1 expression in specimens from 87 patients and found a significant association of SF3B1 expression with progression-free and overall survival. In conclusion, SF3B1 emerges as both a potential prognostic factor and therapeutic target in PDAC, impacting cell proliferation, migration, and apoptosis. These findings warrant future studies on this new therapeutic strategy against PDAC.

## Introduction

In the past decade, the 5-year overall survival rate for individuals with pancreatic ductal adenocarcinoma (PDAC) has increased twofold, rising from 6% to just 13% 1. Nevertheless, there has been a significant increase in the global incidence of PDAC in recent years. Predictions indicate that this tumor type is poised to be the second leading cause of cancer-related deaths within the next 5 years. This grim outlook is attributed to delayed diagnoses and invariably strong resistance to available therapies, underscoring the urgency for research focused on identifying new biomarkers for early detection and investigating more efficacious treatment options for PDAC 2.

Extensive cancer genomics investigations have delineated subtypes in many human malignancies, including breast and lung cancer. These efforts have frequently led to clinically significant strategies aimed at enhancing patient care 3,4.

Comparable studies have been conducted in PDAC, shedding light on pivotal pathogenesis mechanisms. While the identification of "actionable" therapeutic targets in PDAC has yet to be realized, except for PARP1 inhibitors targeting BRCA1/BRCA2 mutations 5, the expanded understanding of the underlying genetics underscores the need for new precision medicine strategies 6.

In the last few years, studies indicated that alterations in the spliceosome machinery may hold a crucial role in PDAC progression. These findings suggest that targeting dysregulated splicing could represent a new strategy to combat PDAC 7.

Alternative splicing (AS) is a fundamental process in eukaryotic gene expression, involving the removal of non-coding intron sequences from pre-mRNAs. This process allows for the selective inclusion or exclusion of specific exons, contributing to the generation of diverse mature mRNAs 8-10. Alternative splicing is achieved by various splicing factors organized into a multi-protein complex termed spliceosome 11. This complex orchestrates the removal of introns and the inclusion or exclusion of specific exons, allowing for the production of multiple transcripts from a single gene. These diverse transcripts can be translated into proteins with distinct functions. Irregularities in splicing factor genes, encompassing alterations in expression levels or the presence of mutations, have the capacity to produce abnormal mRNA splicing patterns on a genome-wide scale 12. This phenomenon is a prevalent characteristic observed in various cancer types, spanning from haematological to solid cancers 13. Numerous splicing factors alterations have been identified in PDAC. Notably, a specific single nucleotide polymorphism in SF3A1 has shown a significant association with the risk of cancer development, particularly in conjunction with environmental factors such as smoking and consumption of alcoholic beverages 14. In patients with PDAC, non-silent mutations in SF3B1 have been reported 15, Additionally, SF3B4 exhibits downregulation in PDAC cells, and its overexpression has been observed to augment cell proliferation and motility 16.

Alterations in splicing also hold prognostic value and make substantial contributions to tumorigenesis 17. A comprehensive genomic study, which included more than 30 cancer types from the Cancer Genome Atlas (TCGA), including PDAC, unveiled thousands of cancer-specific alternative splicing events 18. In the quest for novel prognostic indicators for PDAC, Yu and collaborators recently delved into aberrant splicing patterns, utilizing RNA-sequencing (RNA-seq) data from TCGA and the SpliceSeq databases 19. Their findings indicated a significant association between aberrant alteranative splicing events of various splicing factors genes and overall survival.

Similarly, a study on 43 PDAC tissues, showed that expression of aberrant splice variants predominantly affects extracellular matrix-associated genes and focal adhesion genes, suggesting their role as diagnostic and therapeutic targets 21.

Considering these studies, the dysregulation of splicing is increasingly recognized as a significant characteristic in the pathogenesis of which could represent a novel vulnerability which could be exploited for cancer therapy. The potential of pharmacologically modulating spliceosome activity using small molecules targeting the SF3b complex, such as FR901464, GEX1A, and pladienolides, has been demonstrated in various pre-clinical studied 22. E7107, a pladienolide derivative was tested in a phase 1 trial, showing a stabilization of the disease in 8 out of 40 patients and attained partial-response in one patient with metastatic PDAC. Regrettably, the trial had to be discontinued due to the occurrence of vision impairment in two cases 23. Another similar modulator of splicing, H3B-8800, is under investigation in the NCT02841540 phase 1 trial 24. Furthermore, it was found out that the splicing factor SF3B1 is a substrate of CDK7, a kinase involved in cell cycle regulation and transcription. Indeed, SY-351, a highly selective irreversible CDK7 inhibitor showed a potent effect in splicing modulation 25. Of note, CDK7 has emerged as a target to address chemoresistance, as revealed by a recent kinome-wide CRISPR-Cas9 loss-of-function screening conducted in the PDAC cell line derived from the KPC mouse model 26.

Herein, we explored the potential of targeting altered splicing as an innovative approach in the treatment of PDAC. Our investigation involved the comprehensive characterization of the global splicing landscape in five primary PDAC cell cultures. We assessed the anti-tumor effects of SF3B1 modulators, specifically E7107 and Pladienolide-B, both *in vitro* and *in vivo*, utilizing orthotopic mouse models. Additionally, we determined the levels of expression of SF3B1 in tissues from PDAC patients and established its correlation with clinical outcomes.

## Methods

### Exploring patient samples: Immunohistochemical analysis of SF3B1 expression

Samples from tumors in 87 patients diagnosed with PDAC who underwent surgical procedures at the University Hospital of Pisa were collected after pancreatic-duodenectomy, total- or distal-pancreatectomy, preserved in paraffin and selected for pathological examination. All samples were collected following the patient's written approval, according to the Ethics Committee of Area Vasta Nord Ovest (CEAVNO) protocol# 724. Tissue micro-arrays (TMAs) were built as previously as previously described 27 and SF3B1 expression was analysed by immunohistochemistry using a monoclonal antibody (D221-3, MBL, Japan). SF3B1 scoring (categorized as low or high, according to a scoring system previously outlined 28) was assessed by two researchers, who were blinded to the clinical outcome of the patients. At the time of the surgical procedure, all patients had not undergone any prior treatment. Following surgery, adjuvant treatment with gemcitabine monotherapy was administered, using gemcitabine at a dose of 1000 mg/m^2^/day on days 1, 8, and 15, with a cycle repeated every 4 weeks.

### Drugs targeting splicing

Splicing modulators E7107 were supplied by H3-Biomedicine (Cambridge, USA), while Pladienolide-B was procured from Cayman-Chemical (Ann Arbor, USA).

### Cells and culture conditions

Five (PDAC-1, PDAC-2, PDAC-3, PDAC-4 and PDAC-5) primary PDAC cell cultures were isolated as previously detailed 29,30. Additionally, two non-malignant pancreatic epithelial cell lines (HPNE and HPDE) were sourced from the American Type Culture Collections (ATCC) 31. The cells were cultured in RPMI-1640 medium (Lonza, Switzerland) supplemented with 10% fetal calf serum and maintained at 37°C with 5% CO2. Primary cells were employed until reaching passage number 20.

### Assessment of drug sensitivity, apoptosis, and wound healing

The sulforhodamine B (SRB) assay was employed to evaluate cell viability following exposure to SF3B1 modulators. PDAC-1 and -3 cells were seeded at a density of 3x10^4 cells/well, whereas PDAC-2 and PDAC-5 were seeded at 5x10^4 cells/well, as previously outlined 32. All experiments were performed in triplicates.

For the evaluation of apoptosis PDAC-1, PDAC-3 and PDAC-5 cells were seeded at a density of 50x10^4^ and treated for 48 hours with 10 nM or 25 nM drug concentration. Following fixation in 4% buffered paraformaldehyde, cells were incubated with 8 mg/ml bisbenzimide-HCl for 20 minutes. Subsequently, cells were examined using fluorescence microscopy (Leica, Germany). A total of 350 cells, randomly selected from microscopic fields, underwent evaluation to ascertain the apoptotic index. This index reflects the percentage of cells exhibiting chromatin condensation and nuclear fragmentation in relation to the overall cell count.

In the wound-healing assay, PDAC-1, PDAC-3, and PDAC-5 cells were seeded at a density of 15x10^4 cells/well and subjected to testing according to previously described procedures 28. Two drug concentrations, 10 nM and 30 nM, were employed. The calculation of the "percentage of migrated cells compared to T=0" for each timepoint involved the formula: (Average scratch area at time Tn-T0)/(Average scratch area at time T0)x100. This calculation provides a measure of the migration of cells at different time points (Tn) compared to the initial time point (T0).

### Integration of RNA Sequencing, rMATS Analysis for Differential Splicing, and RT-PCR

Total RNA was obtained from both PDAC and normal epithelial pancreatic cells using the RNeasy mini-Kit (QIAgen) and underwent processing as detailed in previous protocols 28-33. In short, cDNA sequencing libraries were crafted using the Illumina TruSeq Stranded mRNA Library Prep-LT along with Agencount AMPureXP beads. Subsequently, single-end 100 bp-reads were generated using the HT-v4-SR100 Chip across 8 lanes on the Illumina HiSeq 2500 System, followed by quality check.

The identification of unique AS events, including exon skipping (ES), alternative 5' splice site (A5SS), alternative 3' splice site (A3SS), and retained introns (RI), excluding mutually exclusive exons, was performed using rMATS version 3.2.5 34. Default parameters were applied, and a significance cutoff of FDR < 0.05 was employed. For the analysis of genes affected by alternative splicing, the enrichment tool gProfiler 35 version r1741_e90_eg37 was utilized. This involved pathway analysis using Gene Ontology terms as a source database. To complement this analysis, end-point RT-PCR analysis of treated PDAC cells was executed following previously established procedures 28. The primers sequences are detailed in **Supplemental Table 1**.

### Utilization of an orthotopic mouse model and administration of E7107 treatment

Female nu/nu mice were procured from Harlan Laboratories (Madison, WI, USA). The animal experiments conducted were in compliance with the European Community Council Directive 2010/63/EU for laboratory animal care and adhered to the Dutch Law on animal experimentation.

The procedures involving PDAC xenografts followed a validated working protocol and were approved by the local committee on animal experimentation at the Amsterdam University Medical Centers (DEC HEMA14-01). To be specific, orthotopic injection of 1x10^6 PDAC-5 cells was performed, as described previously 30. One week post-tumor induction, mice underwent PET-MRI scans: [18F]-FDG was intravitreally injected at a dose of 5 MBq per mouse, and imaging was conducted using nanoPET-MRI (Mediso, Hungary). The acquired data and images were subsequently normalized and analyzed following established procedures as described 28-36.

### *In vivo* examinations of E7107 and evaluation of tumor burden through flow cytometry

After inducing tumors, a daily intraperitoneal dose of 2.5 mg/kg E7107 was given for 4 consecutive days, starting one-week post-tumor induction. The control group received a vehicle composed of PBS, 10% ethanol, and 5% Tween-80. Mice were monitored for two weeks before being sacrificed. Tumor tissues were dissociated using gentleMACS™ C Tubes and Falcon 40 µm Cell Strainer to obtain single-cell suspensions. The samples were initially stained with 7-AAD, washed with FACS buffer (PBS, 0.1% HSA, 0.05%), and then stained with FITC-labeled Mouse Anti-Human CD44 and APC-labeled Mouse Anti-Human CD24 (BD Bioscience, USA). After additional washing and resuspension in FACS buffer, 50 μl of Flow-CountTM Fluorospheres were added to each sample. Fluorescence was measured using a FACS Fortessa flow cytometer, and the analysis was conducted using FACS Diva software.

### Statistics

Statistical analysis and visualization for clinical outcomes involved correlating them with N-stage, grade, resection margins, and SF3B1 expression through univariate analysis, employing the Mantel-Cox (Log-rank, Chi-Square) test. Survival rates were calculated using the Log-Rank test and depicted using the Kaplan-Meier method. To assess the prognostic relevance a multivariate model was constructed, selecting independent prognostic factors among all clinicopathological parameters (considered as covariates) using the backward stepwise elimination (Wald) method. The statistical analysis of clinical and preclinical data was executed using SPSS version 23 (IBM, USA) and GraphPad Prism version 9.

## Results

### Primary PDAC cultures exhibit modified splicing profiles and responsiveness to splicing modulators

**Figure 1:**
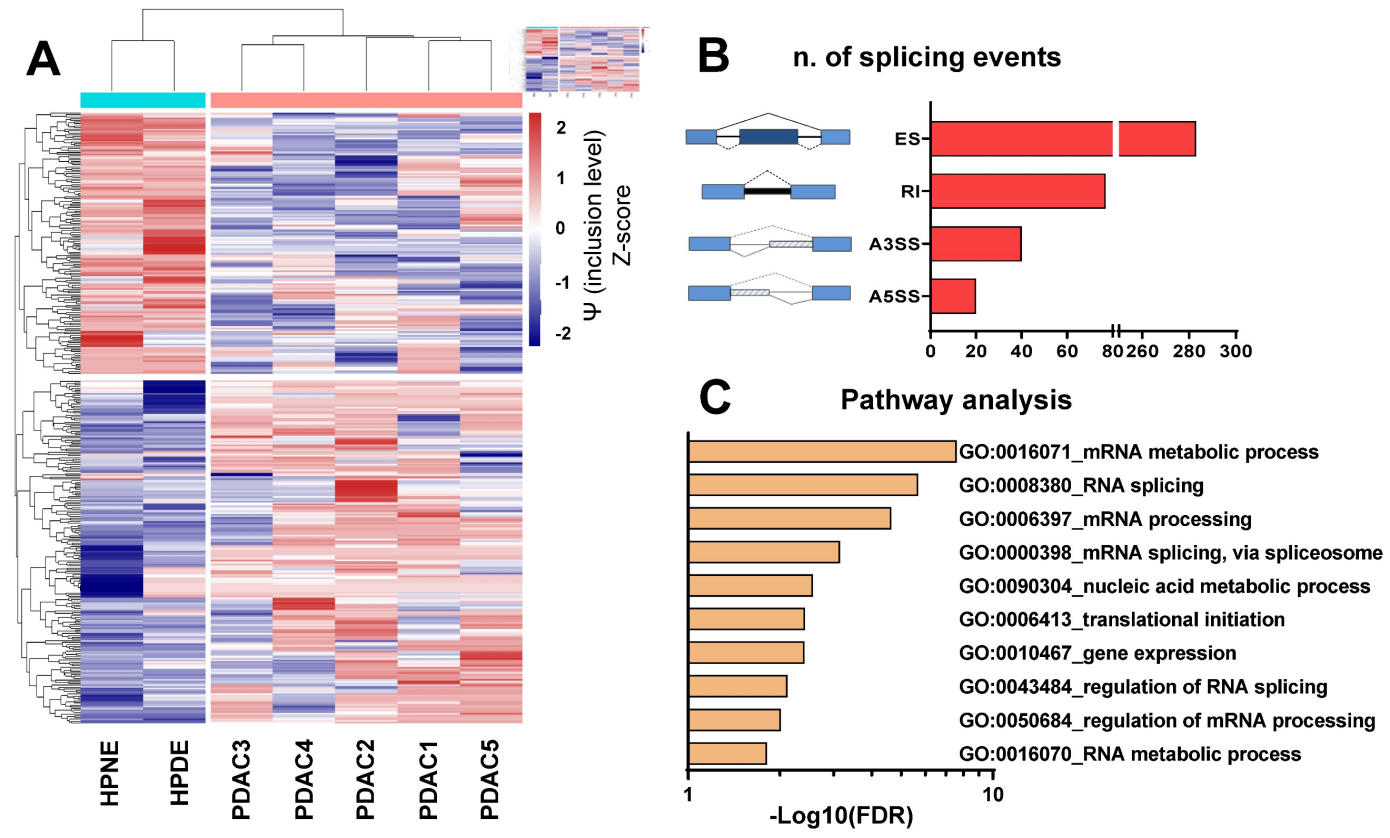
The figure presents the findings of the differential splicing analysis comparing five PDAC cultures with two normal pancreatic cell lines on the basis of transcriptomics data. **(A)** Hierarchical clustering was executed using all (i.e., 420) statistically significant differential splicing occurrences. The incorporation levels, expressed as Percentage Spliced-In (Y) for each event, underwent normalization using Z-scores and were portrayed as a heatmap. The color-coded bar positioned above the heatmap illustrates the phenotype of the analyzed cells, with Cyan signifying normal cells and Red indicating PDAC. **(B)** Illustration outlining various alternative splicing event types as identified by the rMATS algorithm. Exon skipping (ES) are illustrated showing a blue exon which can be excluded from the mRNA transcript); Retained intron (RI) are illustrated showing a black line which indicates the included intron in the mRNA); Alternative 3' splice site (A3SS) and Alternative 5' splice site (A5SS) are illustrated showing boxes under striped lines included in the mRNA. The bar graph displays the count of splicing events (FDR<0.05). **(C)** Gene Ontology (GO) enrichment analysis utilizing all genes impacted by noteworthy differential splicing events (FDR<0.05). The bar plot illustrates top 10 GO terms, each encompassing the highest number of genes within that specific term.

Initially, we examined the splicing profiles of five primary cultures previously identified as representative models of PDAC 30-37. To achieve this objective, we conducted a comprehensive whole-transcriptome splicing profile analysis on primary PDAC cells and two non-malignant pancreatic epithelial cell lines (HPNE and HPDE). Our analysis identified 420 significant differential splicing events (**Figure 1A-B**), with 340 genes predominantly associated with the regulation of mRNA splicing, nucleic acid metabolism and gene expression, as indicated by enrichment analysis on Gene Ontology terms (**Figure 1C**, **Supplemental Table 2**). Guided by the hypothesis that altered splicing could represent a vulnerability in PDAC cells, we assessed the impact of SF3B1 modulators on both cell proliferation and migration.

The selection of SF3B1 as a candidate target in our analysis was based upon existing literature highlighting the significance of SF3B1 alterations in various types of cancers 38. SF3B1 serves as a crucial component within the spliceosome machinery and stands out as the most commonly mutated splicing factor observed across various cancers, including PDAC 15. Remarkably, the significance of SF3B1 extends beyond its mutation, as numerous studies have demonstrated a clear link between changes in SF3B1 expression levels and the aggressiveness of various cancers, including prostate cancer, hepatocarcinoma, and diffuse malignant peritoneal mesothelioma 28-40. Furthermore, our decision was supported by the evidence of potent cytotoxic activity exhibited by SF3B1 modulators (Meayamycin-B, Pladienolide-B, and E7107) *in vitro*, as indicated by their low nanomolar IC50 values 28-41. These findings underscore the potential therapeutic impact of targeting SF3B1 in the context of cancer. Lastly, the potential therapeutic significance of SF3B1 alterations is underscored by the fact that drugs targeting SF3B1 have entered clinical trials 23,24. In light of these compelling studies, we considered SF3B1 as a promising target candidate for investigation in our PDAC models and in PDAC tissues.

**Figure 2:**
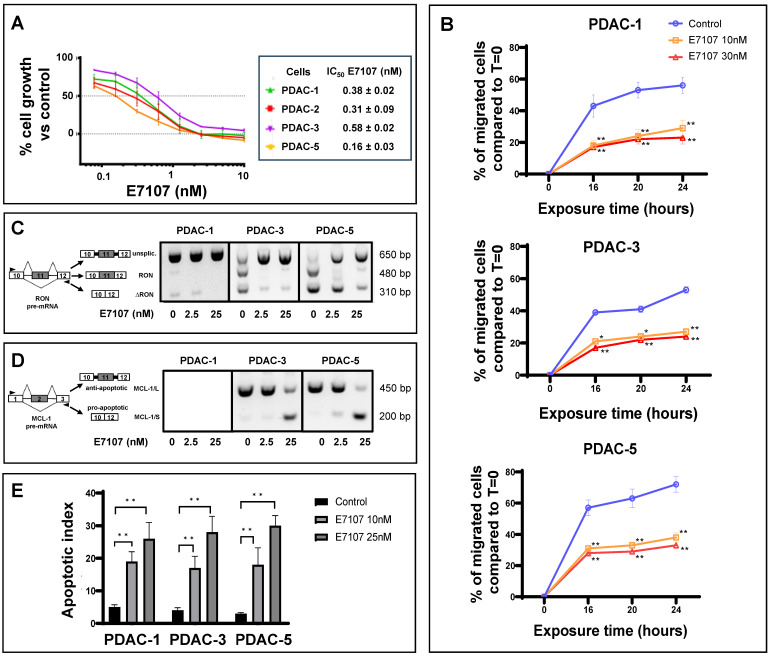
** Impact of the splicing modulator E7107 on PDAC cell growth, migration and apoptosis. (A)** Inhibition of cell proliferation. In four primary cell cultures (PDAC-1, PDAC-2, PDAC-3, and PDAC-5), E7107 induced a dose-dependent suppression of cell proliferation. Cell viability, assessed using the SRB assay, was determined after a 72-hour exposure to escalating concentrations of E7107. The IC50 values, indicating the drug concentration that inhibits 50% of cell growth, were determined by graphically interpolating dose-response curves and are presented in the associated table. PDAC-4 cells were excluded from testing due to suboptimal growth patterns. **(B)** Inhibition of migration. A wound-healing test was conducted on PDAC-1, PDAC-3, and PDAC-5 cells, which were incubated with 10 and 30 nM E7107 for 16/20/24 hours following the introduction of wound tracks. The data depict the percentage of cells migrated within the wound track, presented as mean ± SEM from three independent experiments, with a minimum of six wells per condition in each experiment. Statistical significance compared to control cells at each timepoint was highlighted by asterisks, as follows: *p=0.05, and **p=0.01, Student's t-test. **(C)** Modulation of splicing profiles of RON. On the right panel, representative images of PCR analysis for RON in PDAC-1, PDAC-3, and PDAC-5 cells are displayed after 24 hours of incubation with 2.5 and 25 nM E7107. On the left panel, schematics illustrating pre-mRNA structures are presented, indicating primer annealing sites with black arrows and showcasing the predicted PCR products. **(D)** Modulation of splicing profiles of MCL-1. On the right panel, representative images of PCR analysis for MCL-1 in PDAC-1, PDAC-3, and PDAC-5 cells are displayed after 24 hours of incubation with 2.5 and 25 nM E7107. On the left panel, schematics illustrating pre-mRNA structures are presented, indicating primer annealing sites with black arrows and showcasing the predicted PCR products. **(E)** Induction of apoptosis. The apoptotic index of PDAC-1, PDAC-3, and PDAC-5 cells was determined following treatment with E7107 (10 and 25 nM). The presented data are expressed as mean ± SEM derived from three independent experiments performed in duplicate.

All PDAC cultures exhibited high sensitivity to both E7107 and Pladienolide-B exposure, with IC50 values falling within the low nanomolar range (depicted in **Figure 2A** and **Supplemental Figure 1A**). Given that one of the main challenges in PDAC management lies in its early local/systemic dissemination, we investigated the impact of E7107 on PDAC cell migration. Employing a 24-hour wound-healing assay on the two relatively more drug-resistant (PDAC-1 and PDAC-3) and the more sensitive (PDAC-5) cells, we demonstrated that exposure to 10 and 30 nM E7107, as well as Pladienolide-B, led to a notable suppression of migration compared to controls across all examined time points (**Figure 2B**, **Supplemental Figure 1B**).

Interestingly, this effect was linked to the mis-splicing of the proto-oncogene RON. The PDAC cultures expressed indeed the truncated variant ∆RON that has a critical role in driving cancer cell motility owing to its persistently activated activated kinase function 42,43. Exposing the cells to 2.5 and 25 nM E7107 or Pladienolide-B for 24 hours led to intron retention in the RON transcript and a subsequent reduction in transcript abundance, likely attributable to nonsense-mediated decay (**Figure 2C**, **Supplemental Figure 1C**, **Supplemental Figure 2**). Utilizing a comparable PCR approach, we investigated the splicing patterns of MCL-1 through RT-PCR following a 24-hour exposure to 2.5 and 25 nM E7107. The results revealed a distinct induction of the truncated protein isoform MCL-1/S (short) variant (**Figure 2D**), known for its characteristic pro-apoptotic functions 44. Concurrently with the modified splicing of these apoptotic regulators, there was a notable rise in the percentage of apoptotic cells following treatment with both E7107 and Pladienolide-B (**Figure 2E**, **Supplemental Figure 1D**).

### The *in vivo* growth of PDAC tumors is inhibited by E7107

**Figure 3:**
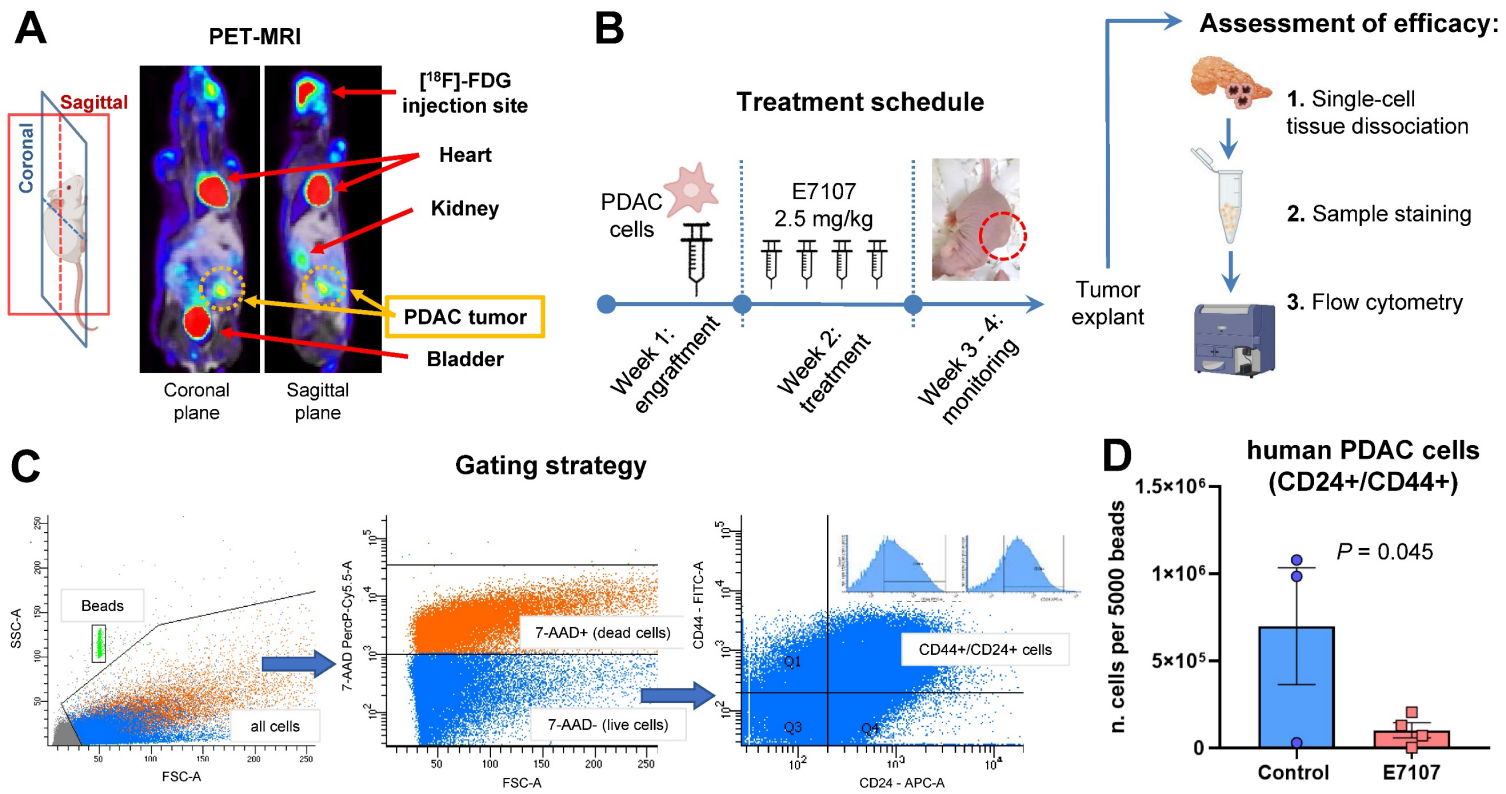
**E7107 hinders the growth of tumors in the orthotopic PDAC-5 model. (A)** Representative PET-MRI of the PDAC-5 orthotopic tumor conducted one week following surgery. **(B)** Experimental design: Mice engrafted with PDAC-5 cells randomly assigned one week after engraftment (four animals per group), were treated with E7107 in four doses (2.5 mg/kg each). The well-being of the animals was observed continuously until the point of sacrifice. Tumor load was assessed through flow cytometry. Neoplastic infiltrates and pancreatic tissues (refer to **Supplemental Figure 3**) were homogenized to generate a single-cell suspension. Subsequently, they were washed and stained with 7AAD to evaluate viability. Antibodies that specifically bind to hCD44 (labeled with FITC) and hCD24 (labeled with APC) were employed. **(C)** Gating strategy: Events were obtained by enumerating 5000 beads for each sample. Human PDAC cells deemed viable (7-AAD-negative) with both surface markers, namely hCD44 and hCD24 were distinguished from murine cells. **(D)** Evaluation of tumor burden in E7107-treated mice conducted in comparison to controls. The bar graph illustrates the count of human tumor cells measured for every 5000 beads for sample (analyzed using Student's t-test). A single data point from the control group was omitted because of a technical error.

Orthotopic mouse models were created through the injection of tumor cells into the pancreas of nu/nu immune-deficient animals (**Figure 3A**).

PDAC-5 cells were chosen for *in vivo* studies after analyses of the effects of E7107 involved in spheroid models of PDAC-1, PDAC-3, and PDAC-5 cultures. These studies revealed a significant disruption of spheroids, with the lowest spheroid area observed in PDAC-5 cells upon treatment with E7107, as illustrated in **Supplemental Figure 2**.

A week later, E7107 was given intraperitoneally based on the treatment schedule (depicted in **Figure 3B**), and the mice were monitored over a three-week period until the control mice exhibited tumor masses in the vicinity of the pancreas. The effectiveness of E7107 was evaluated on *ex-vivo* explanted tumors by quantifying the presence of PDAC-5 cells using flow flow cytometric analysis of CD24 and CD44 45,46 (**Figure 3C**, **Supplemental Figure 3A**). Animals treated with E7107 displayed a significantly reduced number of cancer cells compared to untreated animals (**Figure 3D**). Simultaneous assessment of the mice's weight indicated comparable values in both the treated and control groups, implying that this treatment did not elicit any signs of toxicity (**Supplemental Figure 3B**).

### Elevated expression of SF3B1 serves as an adverse prognostic factor in PDAC

SF3B1 exhibits overexpression across various tumor types 47,48 including PDAC as demonstrated through the examination of of RNA sequencing expression data of 179 PDAC and 332 normal pancreatic tissues from the TCGA and GTEx projects (http://gepia.cancer-pku.cn/detail.php?gene=SF3B1), reported in **Supplemental Figure 4**. However, here we report for the first time the analysis of the protein levels of SF3B1 in a large cohort of PDAC patients and the correlation with the clinical outcome. For this purpose, we established tissue TMAs using paraffin-embedded histological specimens from 87 patients diagnosed with PDAC, and the expression of SF3B1 was evaluated via immunohistochemistry.

This patient cohort exhibited diverse characteristics across various clinicopathological features, shedding light on the heterogenous composition of individuals with PDAC (**Table 1**) Nearly half of the patients (48%) were aged 65 or younger, while the remaining 52% fell into the over-65 category. The gender distribution revealed a similar balanced representation, with 48% male and 52% female patients. In terms of nodal status, a noteworthy 23% of patients presented with N0 status, indicating no lymph node involvement, while the majority (77%) had N1 involvement. Resection margin analysis disclosed that 75% of patients achieved R0 status, while 25% had R1 status, suggesting the presence of residual disease at the margins. Tumor grade distribution showcased a prevalence of 57% for grades 1 or 2, denoting well-differentiated or moderately-differentiated tumors, and 43% for grade 3 tumors, indicating poorly-differentiated tumors.

**Figure 4:**
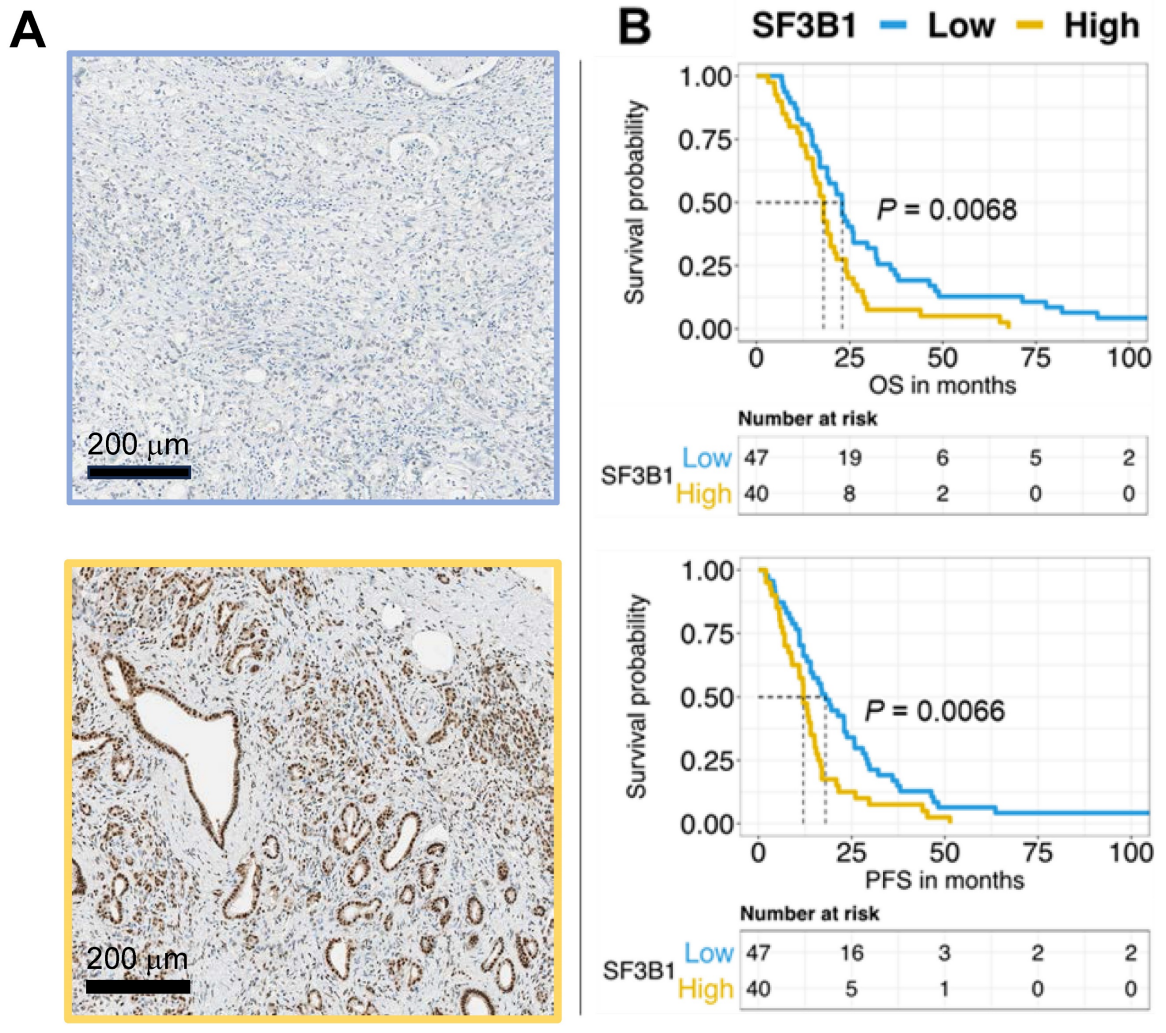
The expression of the SF3B1 protein independently predicts the outcome in patients with pancreatic ductal adenocarcinoma (PDAC). **(A)** Exemplary images depicting low expression (upper panel) and high expression (lower panel) of SF3B1as assessed by immunohistochemical staining in PDAC slides obtained from TMAs of paraffin-embedded PDAC specimens (original magnification 20×). **(B)** The upper panel displays the survival analysis of SF3B1 protein expression concerning Overall Survival (OS), while the lower panel shows the analysis for Progression-Free Survival (PFS). P-values were assessed through the Log-rank test and illustrated with Kaplan-Meier curves.

The categorization of SF3B1 expression levels showed that 54% of patients had low expression, whereas 46% exhibited high expression. Remarkably, the log-rank test demonstrated the association between elevated SF3B1 levels and reduced overall survival and progression-free survival, with p-values below 0.01. This correlation is visually depicted in the Kaplan-Meier survival curves presented in **Figure 4B**. We confirmed through multivariate analysis the independent prognostic significance of the expression of SF3B1 along with the age of the patients (**Table 1**). However, Chi-square analysis examining potential confounders in relation to SF3B1 expression revealed no significant correlation between SF3B1 expression and age, nor with other clinicopathological features (**Supplemental Table 3**).

## Discussion

In this study, we illustrate that manipulating the essential spliceosomal protein SF3B1 exerts robust anti-proliferative, pro-apoptotic, and anti-migratory effects across a series of PDAC models. This insight stems from in-depth sequencing and evaluation of the splicing profiles within these models. Notably, the functions prominently enriched in our dataset involved the regulation of mRNA processing, specifically pre-mRNA splicing. This aligns with the recognized self-regulatory mechanism of numerous splicing factors 7.

Recent investigations offer compelling evidence regarding the effectiveness of spliceosome inhibitors in PDAC 7. Nonetheless, there is a notable absence of thorough pre-clinical experiments aimed at unravelling the rationale for alternative splicing inhibition. In the current study, initially, we profiled the splicing profile of primary PDAC cells in comparison to normal cells using RNA-seq. Consistent with findings from prior investigations 20, we identified differentially spliced variants, primarily of the exon skipping and retained intron types, influencing genes associated with RNA splicing and cancer cell metabolism. Such findings highlight the extensive splicing deregulation observed in PDAC, providing a plausible opportunity for targeted therapeutic interventions.

E7107 and Pladienolide-B displayed strong *in vitro* anti-cancer effects in primary PDAC cultures. These findings are in accord with earlier nobservations in various malignancies 28, 33. Nevertheless, a prior investigation indicates that nearly 25% of about 120 scrutinized spliceosomal genes exhibited differential expression in pancreatic cancer in comparison to normal pancreas. This emphasizes the significance of alternative splicing in the biology of PDAC 21.

Investigations exploring the structure of U2 snRNP attached to Pladienolide-B and E7107, unveiled that the he configuration the catalytic pocket of SF3B1 in collaboration with PHF5A, is essential. Furthermore, these compounds were found to competitively interact with the pre-mRNA substrate in a dose-dependent manner 49,50. These agents showed encouraging outcomes when used alone in various solid tumors (such as colon, breast, and lung cancer) as well as hematological malignancies, as demonstrated in both *in vitro* and *in vivo* studies 51. Furthermore, the synergistic combinations of sudemycins with other anticancer drugs, such as BCL-2/BCL-XL antagonists like ABT-263 or venetoclax, and the inhibitor of Bruton's tyrosine kinase ibrutinib, demonstrated effectiveness in the treatment of chronic lymphocytic leukemia 52, 53. In mouse xenograft models of adult secondary acute myeloid leukemia, E7107 distinctly reduced the load of leukemic stem cells, while preserving the integrity of normal hematopoietic cells 54. Crucially, the latter, along with additional research, showcased the heightened cytotoxic efficacy of spliceosome modulators on cancer in contrast to normal cells, indicating a plausoble therapeutic window 55. This could be elucidated by the varying expression and functionality of SF3B1 and other splicing factors in normal compared to cancer cells 56. Nevertheless, additional investigations involving cells with SF3B1 KO and exploring the connection between the gene and the protein expression of SF3B1 are needed.

The observed antiproliferative activity was accompanied by the induction of apoptosis, a phenomenon that could be, at least in part, attributed to the increased levels of the MCL-1/S variant. This variant is recognized for its distinct pro-apoptotic functions 44. Notably, our previous investigations have already highlighted the omission of exon 2 from MCL-1, leading to the production of this truncated protein isoform following a 24-hour exposure to Pladienolide-B in mesothelioma cells 28.

Control over migration mechanisms is governed not only by transcription factors but also by alternatively spliced transcripts such as ∆RON 57. RON, a receptor with tyrosine-kinase activity, is a member of the MET family, exerting a crucial function in the oncogenesis of numerous tumors in humans 42,58-61. RON is detected in about 90% of PDAC samples and has been documented to have a pivotal role in the tumorigenesis and progression 62. Furthermore, using multiple molecular approaches, an interesting investigation revealed that RON directly regulates HIF-1α overexpression, the latter of which is a key determinant steering the genes associated with the spread of cancer cells. 63. Notably, in 101 PDACs, there was a pronounced co-expression of RON and HIF-1α. Additionally, in PDAC cells the suppression of RON expression resulted in the elimination of HIF-1α expression. Conversely, the introduction of ectopic RON expression triggered HIF-1α expression, implying a regulatory role of RON on HIF-1α.

Our current findings demonstrate that manipulating SF3b disrupts the splicing pattern of this particular variant, leading to a notable decrease in cell migration. This observation aligns with our earlier results in diffuse malignant peritoneal mesothelioma, where exposure to splicing inhibitors yielded comparable splicing modulation and inhibition of migration 28. Furthermore, analogous outcomes have been noted in prior investigations involving diverse preclinical. In particular, elevated levels of distinct splice variants of MUC4, CXCL12, and RHAMM were identified, linking them to heightened invasiveness and metastatic potential 64,65. While the exact mechanism of action remains unclear, it is plausible that the inhibition of migration and the expression of specific splice variants play a crucial role.

Notably, a recent study provided important evidence supporting the involvement of the splicing factor Quaking (QKI) in promoting a plastic, quasi-mesenchymal phenotype, leading to increased migration, resistance to treatments, and ability to adapt to environmental changes in PDAC cells 66. In this respect, a key headway in the PDAC research concerns the recent definition of two major molecular subtypes known as classical and basal-like phenotypes. Classical PDACs express transcription factors functionally involved in pancreatic development and thus exhibit a favorably improved response to combination regimens and a relatively more favourable prognosis. In contrast, basal-like subtypes express bona fide biomarkers of epithelial-to-mesenchymal transition and are therefore chacterized by unfavorable chemotherapy response and dismal prognosis. However, the authors of the abovementioned study 66 recognized the clearcut necessity to employ orthotopic xenograft mouse models with QKI-depleted PDAC cells in order to thoroughly validate its role, as well as the need to conduct large cohorts of PDAC patients for a comprehensive assessment.

In our investigation, the *in vivo* application of E7107 resulted in a modest yet noteworthy decrease in the tumor load within orthotopic models of PDAC, with no observable signs of toxicity. Nevertheless, a significant drawback in this research was the reliance on a single end-point, emphasizing the need for future investigations incorporating bioluminescent orthotopic models. These models would facilitate continuous monitoring of tumor progression both pre-randomization and post-drug administration 28.

In addition, our examination on an extensive cohort of patients affected by PDAC, uncovered that the high expression of SF3B1 correlates with a markedly unfavorable prognosis. These findings align with our earlier discoveries in malignant mesothelioma samples 28, underscoring the rationale for forthcoming trials that could pave the way for the use of splicing modulators into clinical practice.

In particular, our studies indicate that individuals exhibiting an elevated expression of SF3B1 experience poorer prognosis in contrast to those with lower expression. This is attributed to the widespread splicing disruption. Nevertheless, it also implies a potential increased sensitivity to SF3B1 modulators, presenting a novel precision medicine strategy for more efficacious therapeutic interventions in addressing these exceptionally chemoresistant cancers.

In summary, there is an urgent demand for innovative treatments in addressing PDAC, a lethal tumor marked by invasive behaviour and resistance to existing therapeutic protocols. Our preclinical and clinical findings propose novel prognostic implications for SF3B1, encouraging further studies that could potentially validate and extend strategies targeting spliceosome activity.

## Supplementary Material

Supplementary figures and tables.

## Figures and Tables

**Table 1:** Results from univariate and multivariate Cox regression analyses, exploring clinicopathologic characteristics, clinical outcomes, and SF3B1 protein expression in patients with PDAC.

Univariate analysis		N	OS, months (95% CI)	*P*	PFS, months (95% CI)	*P*
*Age, Years*	≤65	42	21.5 (14.4-28.6)	0.013^*^	15 (12.2-17.8)	0.011^*^
	>65	45	18 (16.1-19.9)		12 (8.7-15.3)	
*Sex*	Male	42	19 (13.1-24.9)	*0.509*	15.5 (8.8-15.3)	*0.181*
	Female	45	19 (13.7-24.3)			
*Nodal Status*	N0	20	19.5 (6.3-32.6)	*0.526*	14 (11.4-16.6)	*0.965*
	N1	67	19 (16.7-21.3)		14 (10.4-17.5)	
*Resection Margin*	R0	65	19 (15.7-22.3)	*0.411*	14.7 (11.9-17.5)	*0.399*
	R1	22	19 (13.7-24.3)		13 (10-15)	
*Grade*	1 or 2	50	19 (14.5-23.50)	*0.247*	14 (11.1-16.9)	*0.192*
	3	37	19 (15.6-22.3)		14 (10.3-17.7)	
*SF3B1*	Low	47	23 (20.7-25.3)	0.007^*^	18 (13.3-22.7)	0.007^*^
	High	40	18 (16.0-20.0)		12 (10.3-13.7)	
**Multivariate analysis**	**df**	**Risk of death HR (95% CI)**		***P***	**Risk of relapse HR (95%CI)**	***P***
*Age*	1	1.7 (1.1-2.7)		0.019^*^	1.7 (1.1-2.8)	0.016^*^
*SF3B1*	1	1.8 (1.1-2.8)		0.011^*^	1.8 (1.1-2.7)	0.012^*^

CI, Confidence intervals; df, degrees of freedom; OS, Overall Survival; PFS, Progression free Survival; HR, Hazard ratio; *P<0.05
