# Supplementary material for: Exploring Splicing Modulation as an Innovative Approach to Combat Pancreatic Cancer: SF3B1 Emerges as a Prognostic Indicator and Therapeutic Target
Source: Int J Biol Sci. 2024 Jun 3;20(8):3173–84. doi: 10.7150/ijbs.92671 (PMC11186358; doi:10.7150/ijbs.92671)
Supplement: Supplementary file 1 — Supplementary figures and tables. [file ijbsv20p3173s1.pdf]

Supplemental Figures

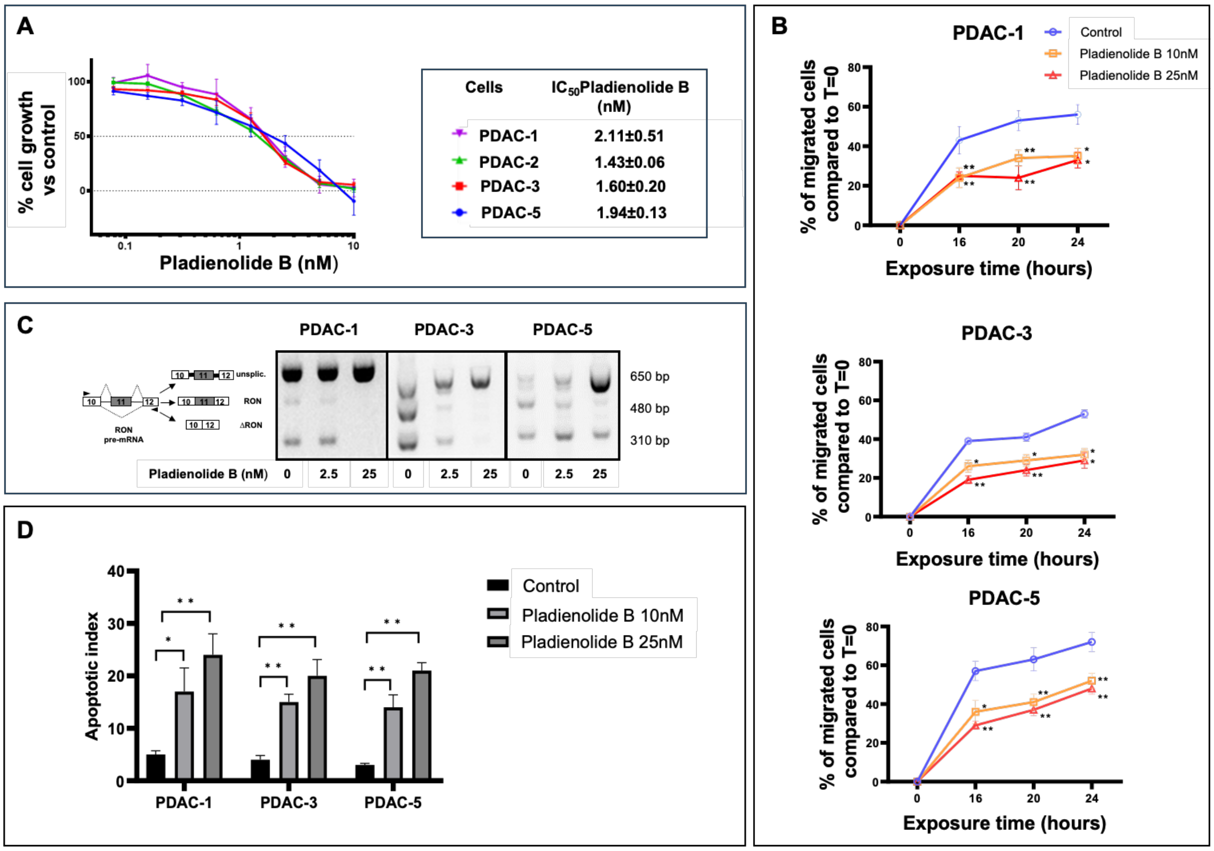

Supplemental Figure 1. Impact of Pladienolide B on PDAC cell growth, migration and apoptosis. **(A)** Inhibition of cell proliferation. In four primary cell cultures (PDAC-1, PDAC-2, PDAC-3, and PDAC-5), Pladienolide B induced a dose-dependent suppression of cell proliferation. Cell viability, assessed using the SRB assay, was determined after a 72-hour exposure to escalating concentrations of Pladienolide B. The IC50 values, indicating the drug concentration that inhibits 50% of cell growth, were determined by graphically interpolating dose-response curves and are presented in the associated table.

(B) Inhibition of migration. A wound-healing test was conducted on PDAC-1, PDAC-3, and PDAC-5 cells, which were incubated with 10 and 30 nM Pladienolide B for 16/20/24 hours following the introduction of wound tracks. The data depict the percentage of cells migrated within the wound track, presented as mean ± SEM from three independent experiments, with a minimum of six wells per condition in each experiment. Statistical significance compared to control cells at each timepoint was highlighted by asterisks, as follows: *p=0.05, and **p=0.01, Student's t-test.

(C) Modulation of splicing profiles of RON. On the right panel, representative images of PCR analysis for RON in PDAC-1, PDAC-3, and PDAC-5 cells are displayed after 24 hours of incubation with 2.5 and 25 nM Pladienolide B. On the left panel, schematics illustrating pre-mRNA structures are presented, indicating primer annealing sites with black arrows and showcasing the predicted PCR products.

(D) Induction of apoptosis. The apoptotic index of PDAC-1, PDAC-3, and PDAC-5 cells was determined following treatment with Pladienolide B (10 and 25nM).

The presented data are expressed as mean ± SEM derived from three independent experiments performed in duplicate.

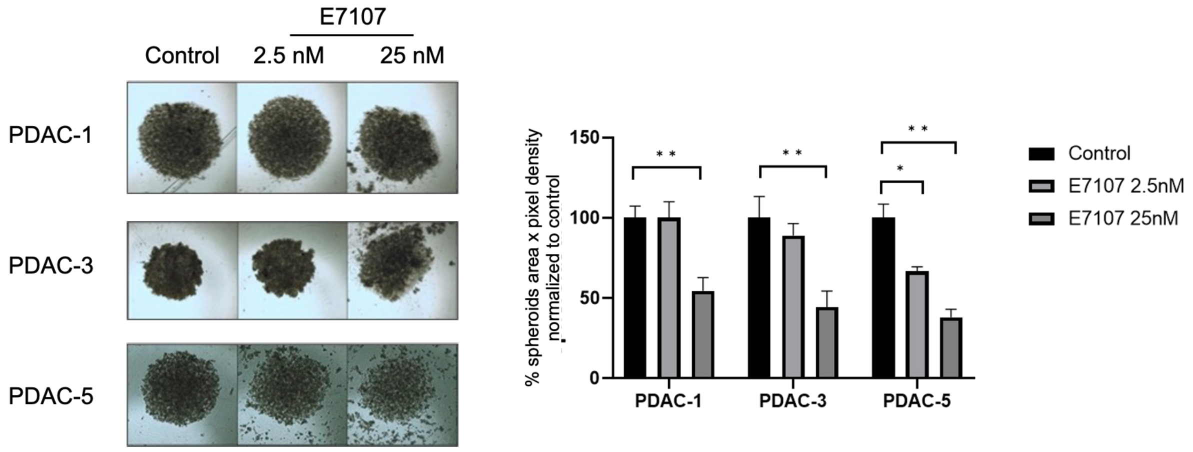

Supplemental Figure 2. Evaluation of the effects of E71017 on PDAC spheroids. The spheroids were generated in plates coated with a layer of agarose. After 3 days, spheroids exceeding a diameter of 200 µm underwent treatment with 2.5 or 25 nM E7107 for 7 days. Automated phase-contrast microscope images of the spheroids were analyzed by ImageJ (NIH, Bethesda, USA) and expressed as mean spheroid area values, as described previously (26)

Representative images of PDAC-1, PDAC-3, and PDAC-5 spheroids following treatment with 2.5 and 25 nM E71017 are presented on the left. The bars on the right depict the percentages in comparison to controls, calculated using the mean of spheroid area multiplied by pixel density ± SEM, with statistical significance indicated as $*P<0.01$ and $**P<0.05$.

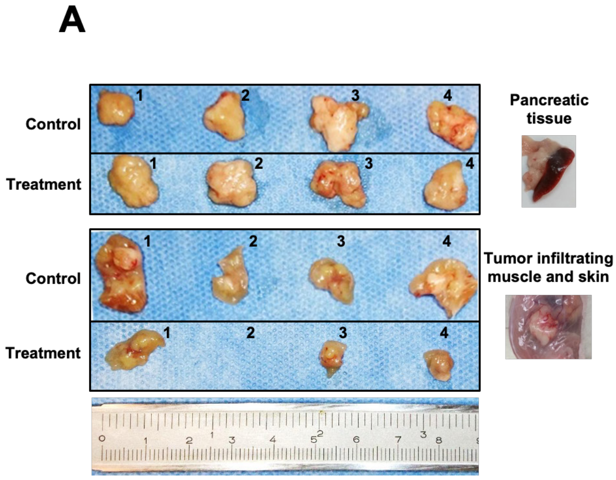

B

	Average weight (g)	
**Day**	**Control**	**E7107**
1 (start treatment)	20.8±1.6	21.6±2.3
2	21.4±1.6	21.2±2.2
3	20.8±1.6	20.2±2.3
4	21.2±1.6	19.8±2.1
7	21.7±1.5	20.5±2.2
14	21.4±1.5	22.3±2.0
21	22.4±1.9	23.2±2.1

Supplemental Figure 3. (A) Examination of resected tumors was conducted before the dissociation into single cells for FACS. The visual inspection includes pancreatic tissue as well as muscle and skin surrounding the surgical site where tumor infiltration occurred. (B) The mean weight of mice ± standard deviation is presented for both the group treated with E7107 and the control animals.

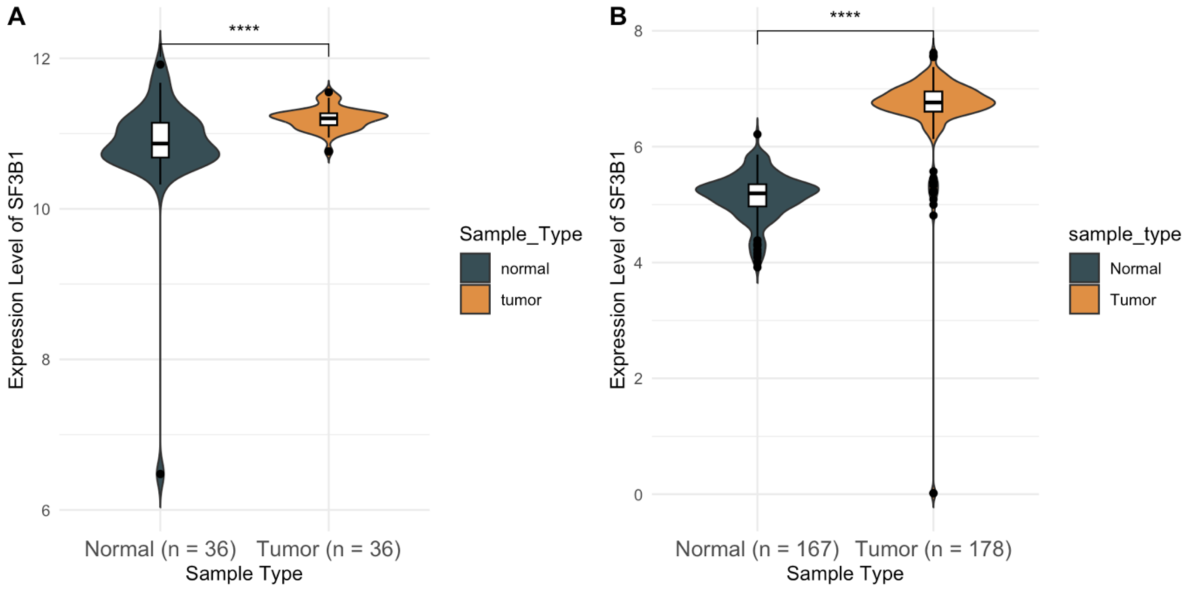

Supplemental Figure 4. Analysis of SF3B1 expression in pancreatic normal and cancer tissues using RNA sequencing data. Violin plots created with library(ggplot2) illustrate SF3B1 expression levels, indicating higher expression in pancreatic tumor tissues than in normal tissues. (**A**) Expression levels of SF3B1 in 36 pancreatic cancer tumor tissue samples and 36 normal tissue samples from the Gene Expression Omnibus (GEO) database GSE15471. (**B**) Expression levels of SF3B1 in 167 normal tissue samples and 178 tumor tissue samples by analyzing the datasets from The Cancer Genome Atlas (TCGA) through the Genomic Data Commons (GDC) Data Portal and Genotype-Tissue Expression (GTEx) databases. Of note, due to limited normal tissue samples in TCGA, GTEx data complemented TCGA data for comparison. Normal tissue samples are represented by dark green bars, and tumor tissue samples are represented by orange bars. The y-axis denotes SF3B1 expression levels. Statistical significance was determined using the Wilcoxon test ($**** : p \leq 0.0001$).

Supplemental Tables

Supplemental Table 1. Primers and melting temperature (Tm) of Mcl-1 and RON

Gene	Primers	Tm
**Mcl-1**	Fw (5’-GCCAAGGACACAAAGCCAAT-3’)<br>Rev (5’-GCTCCTACTCCAGCAACACC-3’)	60°
**RON**	Ex10_Fw (5’-CCTGAATATGTGGTCCGAGACCCCCAG-3’)<br>Ex12_Rev (5’-CTAGCTGCTTCCTCCGCCACCAGTA-3’)	62°

Supplemental Table 2. Significant differential splicing events detected by rMATS analysis (FDR<0.05).

excel file sent to the Editor

Supplemental Table 3. Chi-square analysis of clinical variables and SF3B1 expression

**Variables**	**SF3B1 Expression**		**P-value***
	**Low**	**High**	
**Age**			0.573
≤ 65 Years	24	18	
> 65 Years	23	22	
**Sex**			0.894
Male	23	19	
Female	24	21	
**Nodal Status**			0.356
N0	9	11	
N1	38	29	
**Resection Margin**			0.153
R0	38	27	
R1	9	13	
**Grade**			0.387
Grade 1 and 2	29	21	
Grade 3	18	19	

Notes: All variables were tested by Chi-Square test; P-value <0.05 is considered as significant; Grade refers to histological grade; Resection margin: R0 = No cancer cells at resection margin, R1 = Cancer cells detected at resection margin; Nodal Status: N0 = no no lymph node involvement, N1 = cancer cells detected in lymph nodes.
